# Accelerated oxidative aging tests for white wines: First correlation between physico-chemical and sensory oxidation responses

**DOI:** 10.1016/j.fochx.2026.103687

**Published:** 2026-02-18

**Authors:** Remy Romanet, Jordi Ballester, Jérôme Mallard, Régis D. Gougeon, Maria Nikolantonaki

**Affiliations:** a Université Bourgogne Europe, Institut Agro, INRAE, UMR PAM, Institut Universitaire de la Vigne et du Vin-Jules Guyot, 21000 Dijon, France; b DIVVA Platform, Institut Universitaire de la Vigne et du Vin-Jules Guyot, 21000 Dijon, France; c Université Bourgogne Europe, Institut Agro, CNRS, INRAE, UMR CSGA, 21000 Dijon, France

**Keywords:** Chardonnay, Accelerated aging, Wine shelf life, Sensory analysis, Wine oxidation, Oxidative stability

## Abstract

Oxidative stability during bottle aging strongly affects white wine sensory quality and shelf life. This study evaluated accelerated oxidative aging methods to produce graded oxidation and to align sensory oxidation with physicochemical markers. Two approaches were tested: ultrasonic irradiation and hydrogen peroxide (H₂O₂) addition. Ultrasound did not reproduce typical oxidative evolution and generated off-flavors. In contrast, controlled H₂O₂ addition reliably induced oxidation consistent with natural aging, supported by colorimetric (CIELAB), antioxidant (DPPH), and sensory analyses (REDOX scale, RATA). Applied to several Chardonnay wines, the protocol revealed matrix-dependent oxidation responses. Sensory oxidation scores correlated strongly with antioxidant capacity (Ec20) and browning (ΔE), indicating good method robustness. Overall, the H₂O₂-based protocol offers a rapid, practical framework to induce and grade oxidation-related sensory profiles while linking them to antioxidant and color metrics, enabling controlled comparison of oxidative stability across wines.

## Highlights

• First validated protocol simulating white wine oxidation using controlled H₂O₂ stress.
• Combines sensory, oxidative stability, and colorimetric data for multi-dimensional assessment.
• Reveals matrix-specific oxidative responses in Chardonnay wines.
• Strong predictive link between REDOX sensory scores, browning (ΔE), and Ec20.
• Enables rapid screening of white wine oxidative stability and aging potential.

## 1 Introduction

Anticipating the long-term oxidative stability of wines during bottle aging, particularly for dry white wines, is a key challenge for the production of premium wines. This challenge is largely driven by the need to preserve sensory quality over time during shelf life. Gaining insight into oxidative processes requires addressing how chemical oxidation reactions, especially those involving non-volatile compounds (e.g., phenolic condensation products, nucleophilic additions to electrophiles, reactive oxygen species, and their oxidation products such as aldehydes and ketones), affect both the chemical composition and the sensory attributes of wine.

Chemical oxidation in white wines occurs naturally over their shelf life due to the interaction between dissolved molecular oxygen and transition metals (Karbowiak et al., 2019; Nikolantonaki et al., 2019; Roullier-Gall et al., 2019). It can be further promoted by environmental factors such as closure failure, light exposure, and elevated storage temperatures (Karbowiak et al., 2019; Makhotkina et al., 2012; Mattivi et al., 2015; Ugliano, 2013). Sensory manifestations of oxidation include browning of color, loss of fresh fruity and floral aromas, and the development of off odors. Indeed, across varieties and origins, oxidative evolution is recurrently associated with the emergence of notes such as bruised apple, walnut/curry, honeyed and cooked-vegetable/cooked-fruit attributes, which are widely used to describe oxidation progression. While the kinetics and magnitude of oxidation can differ among wines, this robust sensory signature provides a practical reference framework to define and grade oxidation status. These changes are associated with the formation of oxidation products, including aldehydes (e.g., acetaldehyde, phenylacetaldehyde, methional, o-aminoacetophenone, and 3-(methylthio) propionaldehyde), lactones (e.g., sotolon), and norisoprenoids such as trimethyl-1,2-dihydronaphthalene (TDN) (Ballester et al., 2018; Escudero et al., 2002; Ugliano, 2013).

Importantly, the sensory evolution of wine during aging is not linear and is highly dependent on the wine matrix. Initial post-bottling changes may even be favorable, including the dissipation of yeast- or bacterial-derived off-odors and a reduction in sulfur dioxide notes. Over time, however, wines typically lose their fresh character and may develop complex tertiary aromas, referred to as the “aged bouquet.” If this transformation is unfavorable or results in the development of off-odors, the wine's shelf life may be drastically reduced from years and sometimes decades to just months.

To better anticipate and predict wine shelf life, various accelerated aging protocols have been developed and applied in the wine industry. These include oxygen saturation, heating, hydrogen peroxide (H₂O₂) addition, and ultrasonic irradiation (Coppola et al., 2021; Deshaies et al., 2020; Zhang et al., 2016). The objectives of such protocols were to replicate the oxidative changes that occur during long-term aging in a compressed time frame of days or weeks, rather than years.

Oxygen saturation protocols at temperatures ranging from 20 to 35 °C over several weeks to months have been widely used to simulate oxidation-induced browning and the evolution of volatile profiles (Coetzee et al., 2016; Ferreira et al., 1997; Pérez-Magariño et al., 2023; Singleton et al., 1979). These studies report a decline in fruity aromas and overall quality, largely due to the degradation of phenolic compounds and loss of volatile esters and alcohol acetates, key contributors to fruity and floral notes. Additionally, oxidative stress led to the formation of off-flavor compounds, including Strecker aldehydes (e.g., methional, phenylacetaldehyde) and furanic aldehydes (e.g., furfural, 5-hydroxymethylfurfural), which contributed honeyed and overripe aromas.

Heating-based protocols (with or without oxygen control) have been shown to simulate aging more rapidly, typically within 5 to 10 days, at temperatures up to 60 °C. These approaches have helped identify key oxidative markers via GC-O/FPD/MS, including phenylacetaldehyde, methional, and TDN. Their formation is influenced by variables such as oxygen availability, pH, and SO₂ concentration. Comparisons with naturally aged wines (9–12 months) suggested that high-temperature accelerated aging can mimic certain sensory and chemical shifts, although differences in specific markers (e.g., C6-alcohols, β-damascenone) remained.

Ultrasonic irradiation has recently been explored as a tool to accelerate aging in red wines and spirits (Delgado-González et al., 2017; Zhang et al., 2015). High-frequency ultrasound (100 kHz) induced the formation of reactive radicals (e.g., hydroxyethyl and hydroxyl radicals) via water and ethanol oxidation (García et al., 2016; Kubo et al., 2012; Zhang et al., 2015). Most studies to date have focused on enhancing wood compound extraction and mimicking barrel aging. For red wines such as Cabernet Sauvignon, ultrasound treatment has been shown to increase color intensity through phenolic polymerization, suggesting a potential for simulating oxidative aging (Ferraretto & Celotti, 2016). However, to our knowledge, ultrasonic irradiation has not been systematically tested as a method to induce oxidative aging in white wines to a stage suitable for sensory evaluation.

Hydrogen peroxide-based protocols have also shown promise, particularly for red wines (Coppola et al., 2021; Deshaies et al., 2020). When compared to air saturation, H₂O₂-induced oxidation produced similar phenolic transformations and color changes, including anthocyanin degradation and flavanol accumulation. In Syrah red wines, H₂O₂ treatments yielded chemical profiles closer to naturally aged vintages than those aged via heating or laccase-induced oxidation.

For white wines, H₂O₂ combined with heating has been used to study browning and pinking reactions (Minute et al., 2021; Simpson, 1977; Voltea et al., 2022). Oxidation initiated via Fenton chemistry (Fe^2+^/H₂O₂) significantly accelerated browning and phenolic loss, effects measurable by CIEL*a*b* and UV–Vis absorbance (420 nm). These protocols offer a rapid simulation of long-term oxidative changes, but their relevance in terms of sensory attributes remains largely unexplored.

To date, many accelerated aging studies have primarily relied on chemical and/or colorimetric endpoints, while fewer have integrated sensory approaches using standardized oxidation descriptor sets and intensity scales. Because the sensory signature of oxidation in dry white wines is well described in the literature across varieties and origins, this literature-defined sensory framework can be used as a reference to evaluate whether accelerated treatments elicit graded oxidation-type profiles (Ballester et al., 2018; Escudero et al., 2002). Accordingly, the aim of this study was to develop and compare accelerated oxidation approaches for dry white wines, focusing on accelerated aging methodology and establishing a first correlation between accelerated oxidative stress and sensory REDOX evaluation using the literature established sensory framework as reference. Two approaches ultrasonic irradiation and controlled H₂O₂ addition were optimized and compared. Sensory assessment (REDOX oxidation intensity scale and RATA profiling) was combined with physico-chemical measures including DPPH based antioxidant capacity and CIELAB* color metrics, and relationships between these endpoints were examined to quantify correlations between sensory oxidation intensity and physico-chemical, colorimetric evolution under standardized oxidative stress conditions, without claiming prediction of shelf life.

## 2 Materials and methods

### 2.1 Chemicals

1,1-Diphenyl-2-picrylhydrazyl radical (DPPH), citric acid, sodium phosphate dibasic were purchased from Sigma Aldrich (St. Louis, MO, USA). Methanol (99.8%) and hydrogen peroxide 30% weight (H_2_O_2_) were purchased from Chemlab. Ultrapure water comes from a Milli-Q system (Merck, Darmstadt, Germany).

### 2.2 Wine samples

Five dry Chardonnay wines that had undergone complete malolactic fermentation were selected for the study. The wines were fresh commercial Chardonnay wines**,** chosen because they are representative of the grape variety and typical dry white winemaking style after malolactic fermentation. These wines represent a diversity of viticultural regions and winemaking practices and were sourced from four wineries across distinct geographical areas: Hautes Côtes de Beaune (HCB), South Bourgogne (Bourg), Ardèche (Ard), Hautes Côtes de Nuit (HCN), and Chablis Côte de Jouan (ChaJouan). Three of the wines from vintage 2018 and one from 2019 have been used for the development of the accelerated aging protocol (2018-HCB and 2018-Bourg and 2019-HCN). Three additional wines from the 2019 vintage (Ard, HCN, and ChaJouan) were used for validation of the optimized protocol (2019-Ard, 2019-HCN and 2019-ChaJouan). Free sulfites for these wines are given as supplementary Table S1.

### 2.3 Accelerated aging test protocol

#### 2.3.1 Ultrasound irradiation

Wine samples (500 mL) were placed in a TI-H-20 ultrasonic bath (Elma) at 20 °C and irradiated for 3 or 7 days in the dark. Ultrasound was applied at a frequency of 135 kHz and a power output of 250 W. Prior to treatment; all wines were filtered through 0.22 μm membranes to prevent microbial spoilage. Control samples were stored at 4 °C under an argon atmosphere to prevent oxidation. Treatments were performed both with and without oxygen saturation. All experiments were conducted in duplicate.

#### 2.3.2 Chemical oxidation by peroxide addition

Accelerated oxidation was induced by the addition of hydrogen peroxide in concentrations ranging from 0.1 to 1.5 mM, followed by incubation for 8 days at 20 °C in the dark. Control samples were stored at 4 °C under anoxic conditions. As with ultrasound treatments, wines were pre-filtered through 0.22 μm membranes. The H₂O₂ induced oxidation relied on native Fe and Cu content. DO was not monitored because rapid Fenton consumption leads to immediate depletion, making DO minimally informative. H₂O₂ concentrations were calculated based on equimolar reactions with free sulfur dioxide (SO₂), accounting for its neutralization, in accordance with established stoichiometry (Danilewicz, 2007, Danilewicz, 2016). Free sulfites were determined using Sulfilyser. Treatments were conducted in duplicate.

### 2.4 Measurement of wine's antioxidant capacity by DPPH optimized essay

Wine antioxidant capacity was assessed using the DPPH radical scavenging method optimized for white wines (Romanet et al., 2019). A 25 mg/L DPPH solution was prepared in a methanol/citrate-phosphate buffer (60/40 *v*/v, 0.1 M citrate, 0.2 M phosphate, pH 3.6). Increasing wine volumes (0–100 μL) were added to 3.9 mL of DPPH solution to generate calibration curves. To prevent oxidation, all manipulations were carried out under nitrogen in a glovebox. Free SO₂ was removed via CO₂ bubbling (Pegram et al., 2013). Samples were incubated for 4 h in the dark at room temperature and absorbance was measured at 525 nm. Antioxidant capacity was expressed as Ec20, the volume of wine required to reduce the initial DPPH concentration by 20%. The change in antioxidant capacity (∆Ec20; eq1) was calculated as the difference between pre- and post-treatment values.

(1) $$∆{\mathrm{Ec}}_{20}={{\mathrm{Ec}}_{20}}_{\text{aged}}-{{\mathrm{Ec}}_{20}}_{\text{control}}$$

### 2.5 Color determination

CIELAB color parameters (L*, a*, b*) were measured on undiluted wine samples using a CM-5 Konica Minolta spectrophotometer in 5 mL cuvettes. Total color difference (ΔE) between control and aged samples was calculated using the Euclidean formula:

$$∆E=\sqrt{{\left(L_{\text{aged}}^{*}-L_{\text{control}}^{*}\right)}^{2}+{\left(a_{\text{aged}}^{*}-a_{\text{control}}^{*}\right)}^{2}+\left(b_{\text{aged}}^{*}-b_{\text{control}}^{*}\right)}$$

A ΔE > 7 was considered a perceptible color difference in white wines (Quijada Morin et al., 2024).

### 2.6 Sensory analysis

#### 2.6.1 Panel

Sensory analysis took place between November 2018 and March 2021. Three sensory panels were conducted successively during this period, composed respectively of 33 (panel 1), 47 (panel 2) and 13 (panel 3) candidates to participate in sensory sessions. Panelists were recruited among enology students, PhD students, engineers, professors and researchers at the Institute of Vine and Wine at the University of Burgundy in Dijon (IUVV, France). All of them had knowledge and experience on wine tasting, in particular in odor assessment. All participants received an information sheet and provided written informed consent prior to participation. They were informed that their responses would be used exclusively for academic research purposes and that anonymity would be ensured.

#### 2.6.2 Panel training and selection

Panel training was identical for the three panels in three consecutive years. Panelists took part in ten 30-min training sessions to familiarize and generate consensual descriptors related to wine oxidation and reduction odors. They were also trained to quantitatively calibrate their measurements. The training strategy was based on the protocol developed by Ballester et al., 2018. Panelists were first initiated to oxidation and reduction notes in hydro alcoholic solution and then in spiked wines. Afterwards, commercial wines with different ranges of oxidation and reduction intensities as well as ‘clean’ wines (without oxidation or reduction notes) were selected and assessed with the REDOX odor scale. This scale is structured from −5 (strong reduction), to +5 (strong oxidation), and zero (neither reduced nor oxidized) in the midpoint (Ballester et al., 2018).

Panelists were selected based on their discrimination ability, repeatability, and consensus with peers. Final panel composition included 19 (Panel 1: ten females and nine males, average age 24.5), 21 (Panel 2: nine females and twelve males, average age 24.2), and 11 (Panel 3: three females and eight males, average age 29.0) assessors.

#### 2.6.3 Descriptive sensory evaluation

To ensure sample homogeneity and sufficient volume for sensory evaluation, four bottles were pooled per wine prior to treatment. Each treatment condition was prepared in duplicate as independent preparations. For sensory evaluation, duplicate preparations were presented as separate coded samples and evaluated independently. Wines (20 mL) were served at room temperature in black ISO tasting glasses, coded with randomized three-digit numbers and covered with Petri dishes. Panelists rated REDOX intensity and completed a “Rate-All-That-Apply” (RATA) task using 28 descriptors relevant to Chardonnay and wine faults (Butter, Caramel/Vanilla, Grilled, Spicy, Woody, Cork, Dust/Cardboard, Leather/Stable/Gouache, Scotch glue/Varnish, Wax/Naphthalene, Citrus, Exotic fruits, White fruits, Yellow fruits, Honey, Honey/Dried roses, Rancid butter, Bruised apple, Candied/Cooked fruits, Cooked vegetables/Boiled potatoes, Rancio/Madere, Walnut/Curry, Waterlogged, Cabbage/Truffle/Corn, Rotten egg/ Rotten onion, Floral, Hay, Herbs). An open-ended “Other” category was included for unlisted terms.

### 2.7 Statistical analyses

REDOX scores were analyzed using one-way ANOVA (α = 0.05), followed by Fisher's LSD test for post-hoc comparisons. RATA data were first screened via Kruskal–Wallis tests (*p* < 0.05) to identify discriminant descriptors, which were then analyzed by Principal Component Analysis (PCA). Antioxidant capacity data were analyzed using Kruskal–Wallis tests (*p* < 0.05). All analyses were performed in XLStat 2017 (Addinsoft, Paris, France) and Matlab (Mathworks, Natick, MA,USA). Figures were plotted using OriginPro 2021 (OriginLab Corporation, Northampton, MA, USA), SIMCA 2021(Umetrics, Umeå, Sweden) and Matlab (Mathworks, Natick, MA,USA).

## 3 Results and discussion

### 3.1 Optimization of accelerated aging tests

To simulate oxidative conditions in white wines, two experimental accelerated aging strategies ultrasound irradiation and hydrogen peroxide (H₂O₂) addition were tested. Their effectiveness was assessed through sensory REDOX scoring (Panel 1) and antioxidant capacity via the DPPH assay. Sensory profiles from RATA analysis were used to evaluate how closely the treated wines resembled those naturally aged under cellar conditions. Initial dissolved oxygen (DO) levels were considered due to their influence on oxidation mechanisms (Danilewicz, 2003; Waterhouse & Laurie, 2006).

#### 3.1.1 Ultrasound irradiation

The first accelerated aging strategy tested was ultrasound treatment, applied to the 2018-HCB wine. Prior studies had reported positive results using ultrasound in red wines and spirits (Delgado-González et al., 2017; Zhang et al., 2015). Ultrasound irradiation can generate 1-hydroxyethyl radicals via ethanol oxidation, naturally occurring in wine matrices (Elias et al., 2009; Nikolantonaki et al., 2019; Zhang et al., 2015). In our protocol, samples were exposed to ultrasound for 0 (control), 3, or 7 days at constant temperature, power, and frequency, following Zhang et al., 2015.

Table 1 presents the sensory REDOX mean scores and Ec_20_ values as function of time. No statistically significant differences were observed across the treatments in either the REDOX score or antioxidant capacity (Ec20), regardless of whether oxygen saturation was applied (ANOVA, *p* > 0.05). Under anoxic conditions, the initial antioxidant capacity of the 2018-HCB sample was low (Ec20 = 53 ± 2), consistent with literature reports (Romanet et al., 2019). Across all time points (0, 3, and 7 days), no significant variations were detected in either Ec20 or REDOX score. Even when oxygen saturation was applied prior to ultrasound exposure, REDOX scores remained statistically unchanged, and typical oxidative descriptors (e.g., honey, bruised apple, walnut) were not reported by the panel. Instead, panelists frequently noted off-odors described as “plastic,” “burned plastic,” and “chemical” terms cited 28 times for ultrasound-treated samples versus once for the control. These descriptors indicate the emergence of atypical sensory notes under the ultrasound conditions tested, and the underlying origin warrants further investigation. In terms of antioxidant capacity, there was no significant difference between pre- and post-treatment Ec20 values (53 ± 2 vs. 46 ± 3; Kruskal-Wallis, *p* > 0.05). This further supports the conclusion that ultrasound irradiation, under the conditions tested, did not induce a realistic oxidative evolution of white wine. In conclusion, ultrasound irradiation did not yield sensory or oxidative status changes consistent with natural oxidative aging. Instead, it introduced non-characteristic off-flavors, undermining its suitability as a valid accelerated oxidation method for white wines.

**Table 1:** : Sensory evaluation (Redox score) and global antioxidant capacity (DPPH, Ec_20_) of 2018-HCB treated wine with ultrasound irradiations for a total duration of 0, 3 and 7 days under oxygen saturation or not. Statistical analysis has been realized for each oxygen condition according to irradiation duration, using Anova test for Redox score and Kruskal-Wallis test for Ec_20_.

Time (days)	Oxygen saturation	Redox score	p-Value Anova	Ec_20_	p-Value Kruskal-Wallis
0	No	2.7 ± 1.6	0.28	53 ± 2	0.78
3	No	2.9 ± 1.1		50 ± 4	
7	No	2.1 ± 2.3		52 ± 3	
0	Yes	2.2 ± 1.1	0.31	53 ± 2	0.09
3	Yes	1.0 ± 3.0		52 ± 3	
7	Yes	1.5 ± 2.0		46 ± 3	

#### 3.1.2 Chemical oxidation via hydrogen peroxide addition

The second accelerated aging strategy involved initiating chemical oxidation through the controlled addition of hydrogen peroxide (H₂O₂). This approach is supported by prior studies indicating that H₂O₂ contributes to oxidative reactions naturally occurring during wine aging, particularly through its interaction with dissolved oxygen and transition metals (Coppola et al., 2021; Deshaies et al., 2020).

Initial testing was conducted on the 2018-HCB wine, with H₂O₂ concentrations ranging from 0.5 to 7 mM and 1-day incubation. Although results showed some oxidative response, they lacked statistical significance (data not shown). To optimize the method, the 2018-Bourg wine was treated with a fixed H₂O₂ concentration (1 mM) and incubated for 3, 8, or 13 days. Results from sensory analysis (Fig. S1) revealed a significant increase in REDOX scores post-H₂O₂ treatment, regardless of incubation time. The absence of differences among incubation durations led to the selection of 8 days as the optimal aging period, balancing efficacy and experimental manageability. RATA sensory data confirmed that oxidized samples were characterized by descriptors typically associated with wine oxidation, such as honey, walnut/curry, brushed apple or cooked vegetables, while untreated controls retained fruity, floral, and woody characteristics (Fig. S2). These findings supported the potential of H₂O₂ as a reliable agent to simulate oxidation effects comparable to those observed in natural cellar aging.

#### 3.1.3 Optimization of H₂O₂ concentration

**Fig. 1:**
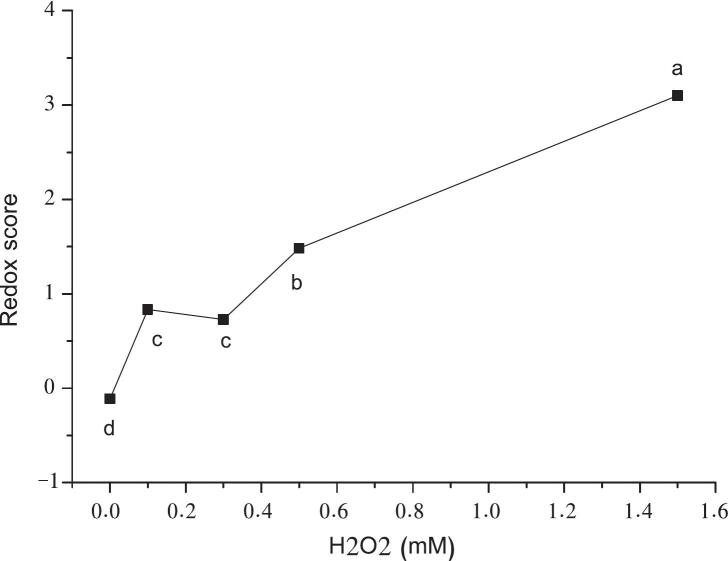
Sensory evaluation (Redox score) of 2019-HCN wine treated with increasing H_2_O_2_ concentrations (0.1, 0.3, 0.5 and 1.5 mM) after 8 days incubation at room temperature before analysis. Letters show significative statistical differences determined by Anova and Fisher LSD test (p < 0.05).

To identify the optimal H₂O₂ concentration, 2019-HCN wine samples were treated with 0.1, 0.3, 0.5 and 1.5 mM H₂O₂ and incubated for 8 days at room temperature. REDOX scores (Fig. 1) showed a statistically significant, concentration-dependent increase (ANOVA, *p* < 0.05), although the correlation was non-linear. This suggests a lag phase in oxidation, likely reflecting the intrinsic antioxidant resistance of the wine matrix. This resistance, specific to each wine, has significant implications. Wines may absorb low levels of oxidative stress without sensory degradation, while higher concentrations surpass protective thresholds, leading to rapid oxidative progression. The identification and characterization of this lag phase is of high scientific interest and needs further exploration in a later time. In summary, our H₂O₂-based protocol appeared to effectively mimic oxidative aging in white wines, both chemically and sensorially. Our results further highlighted the importance of matrix-dependent variability and validate the methodology's suitability for broader application.

### 3.2 Method validation in different white wines

To validate the optimized H₂O₂-based accelerated aging protocol, it was applied to three additional white wines from the 2019 vintage: 2019-HCN**,** 2019-Ard**,** and 2019-ChaJouan. Validation was based on comparisons of antioxidant capacity (Ec20), REDOX score, and color changes after an 8-day treatment at room temperature.

**Fig. 2:**
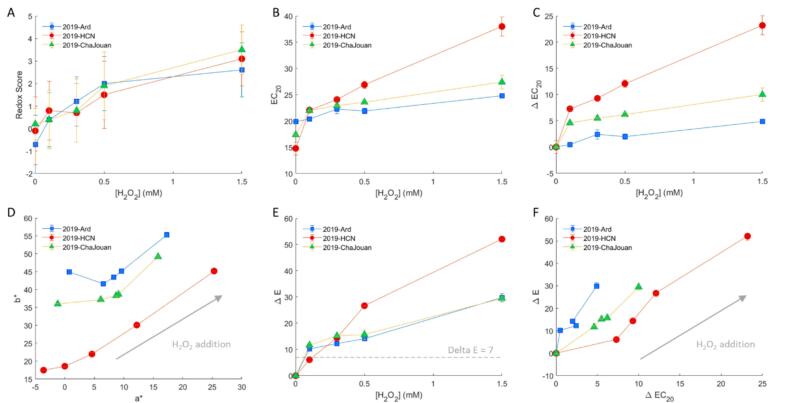
Evolution of the Redox score (A) the antioxidant capacity (Ec_20_) (B) the evolution of antioxidant capacity ΔEC_20_ (C) as a function of H_2_O_2_ concentration added. D represents the evolution of a* and b* parameters from CIELAB analysis according to H_2_O_2_ addition, E represents ΔE as a function of H_2_O_2_ concentration added and F the relation between ΔE and ΔEC_20_. In each plot, 2019-Ard is represented by blue squares, 2019-HCN by red circles and 2019-ChaJouan by green triangles. Error bars correspond to standard deviation calculated from replicates. (For interpretation of the references to color in this figure legend, the reader is referred to the web version of this article.)

Figs. 2B-2C show the evolution of Ec20 values following increasing H₂O₂ additions. All wines exhibited a significant decrease in antioxidant capacity with increasing H₂O₂ concentrations. This decline reflects the consumption of native antioxidants primarily phenolics and sulfur-containing compounds due to oxidative reactions. However, the magnitude of Ec20 change (ΔEc20) varied among wines, revealing intrinsic differences in oxidative resistance. Control samples showed distinct initial Ec20 values (2019-HCN: 14.8 ± 1.2; 2019-ChaJouan: 17.4 ± 0.1; and 2019-Ard: 19.9 ± 0.1), and therefore distinct native oxidative stability. To compare wines oxidative resistance quantitatively, linear regressions were performed using Ec20 as a function of H₂O₂ concentration. The slope (ΔEc20/[H₂O₂]) served as an indicator of oxidative sensitivity. For instance, 2019-HCN had a slope of 13.5 ± 0.8 which involved a rapid loss of antioxidant capacity whereas, 2019-ChaJouan and 2019-Ard were characterized by smaller slopes (5.2 and 3.1 respectively) suggesting higher oxidative resistance (Fig. 2C). Despite having the highest initial antioxidant capacity, 2019-HCN was the most susceptible to oxidation, losing 157% of its antioxidant capacity after 1.5 mM H₂O₂ treatment (ΔEc20 = 23.2). In contrast, 2019-Ard and 2019-ChaJouan showed higher stability (ΔEc20 = 4.9 and 10, respectively). These results support findings from Romanet et al., 2023, suggesting that transient Ec20 alone is insufficient to predict oxidative stability. The wine matrix, including nucleophilic components and other redox-active molecules, must also be considered.

H₂O₂ additions significantly increased REDOX scores in all three wines (ANOVA, *p* < 0.05), indicating perceptible oxidation (Fig. 2A). Scores ranged from 0.4 in controls to 3.5 at 1.5 mM H₂O₂. An exponential regression model showed strong fit and narrow confidence intervals, suggesting reproducible oxidative behavior across different matrices (Fig. S3). While REDOX scores consistently increased, antioxidant capacity (Ec20) responses varied more between samples, highlighting the greater sensitivity of sensory evaluation to oxidative effects. Pearson correlation analysis showed significant links between REDOX score and H₂O₂ (*r* = 0.91, *p* < 0.05) and between Ec20 and H₂O₂ (*r* = 0.77, p < 0.05) and REDOX score vs. Ec20: *r* = 0.75. This confirmed a strong relationship between chemical oxidation and sensory perception.

Color evolution was assessed using CIELAB parameters (Figs. 2D–2F). H₂O₂ additions caused a decrease in lightness (L*, data not shown) and an increase in both a* (green–red) and b* (blue–yellow), consistent with browning (Minute et al., 2021). ΔE values, representing total color difference, surpassed the perception threshold (>7) even at low H₂O₂ concentrations, particularly for 2019-HCN. At 1.5 mM H₂O₂, 2019-HCN showed a ΔE of 52.1 ± 0.0; 2019-ChaJouan a ΔE = of 29.5 ± 0.6 and 2019-Ard a ΔE of 29.8 ± 1.6. ΔE correlated strongly with ΔEc20 (*r* = 0.87, *p* < 0.05), supporting the conclusion that color change reflects oxidative degradation and loss of antioxidant protection. Wines with higher resistance (e.g., 2019-Ard) showed smaller changes for both parameters. Future studies may integrate metal (Fe/Cu) quantification and DO measurements during H₂O₂ oxidation to refine mechanistic understanding**.**

### 3.3 Impact on sensory profile

To characterize the sensory evolution of wines during accelerated oxidation, RATA descriptors with significant sample effects were analyzed using PCA followed by hierarchical cluster analysis (HCA). These analyses were conducted separately for each wine.

Across the three wine matrices, Kruskal-Wallis test (*p* < 0.05) showed that only three descriptors were discriminant for all wines: cooked vegetables / boiled potatoes; walnut / curry and bruised apple, Table 2. These are well-known markers of white wine oxidation (Ballester et al., 2018; Escudero et al., 2002; Silva Ferreira et al., 2002). Four additional descriptors were shared by two wines, 1 related to oxidation (Wax/Naphthalene), and 1 to reductive notes (Cabbage/Truffle/Corn, Rotten egg/ rotten onion) and 1 to fruitiness (exotic fruits). Ten descriptors were unique to individual wine matrices, including scotch glue/varnish, wood, floral, yellow fruits, white fruits, herbs, waterlogged, rancio/madere, candied/cooked fruits and honey/overblown roses, illustrating the chemical and sensory diversity among the studied wines.

**Table 2:** *p*-Value obtained by one-way Anova for the Redox Score and by Kruskal-Wallis test for the 28 sensory descriptors according to H_2_O_2_ addition for the 3 studied wines. Significative p-Value (*p* < 0.05) are in bold.

Descriptor	2019-HCN	2019-Ard	2019-ChaJouan
REDOX Score	**1.15E-17**	**2.84E-16**	**5.73E-13**
Citrus	0.051	0.208	0.081
Butter	0.412	0.182	0.47
Rancid butter	0.686	0.378	0.487
Woody	0.979	0.014	0.396
Cork	0.406	1	1
Caramel / Vanilla	0.178	0.918	0.818
Cabbage / Truffle / Corn	**0.038**	**1.68E-04**	0.248
Wax / Naphthalene	**0.009**	**0.015**	0.195
Scotch glue / Varnish	0.251	0.554	**0.016**
Leather / Stable / Gouache	0.555	1	1
Waterlogged	**0.011**	**0.066**	0.461
Spicy	0.081	1	0.191
Floral	0.316	0.058	**0.001**
Hay	0.245	1	0.553
White fruits	0.445	0.562	**0.004**
Candied / Cooked fruits	0.106	0.94	**0.002**
Exotic fruits	0.181	**0.016**	**0.013**
Yellow fruits	0.861	0.124	**0.05**
Grilled	0.251	0.089	0.465
Herbs	0.555	0.406	**0.013**
Cooked vegetables / Boiled potatoes	**0.022**	**0.034**	**0.001**
Honey	0.835	0.475	0.127
Honey/Dried roses	0.255	**0.009**	0.378
Walnut/Curry	**6.50E-09**	**7.45E-05**	**1.72E-12**
Rotten egg/ Rotten onion	**0.019**	**0.011**	0.132
Bruised apple	**1.36E-06**	**9.76E-05**	**9.22E-05**
Dust / Cardboard	0.069	0.406	0.553
Rancio / Madere	**1.14E-06**	0.224	0.089

**Fig. 3:**
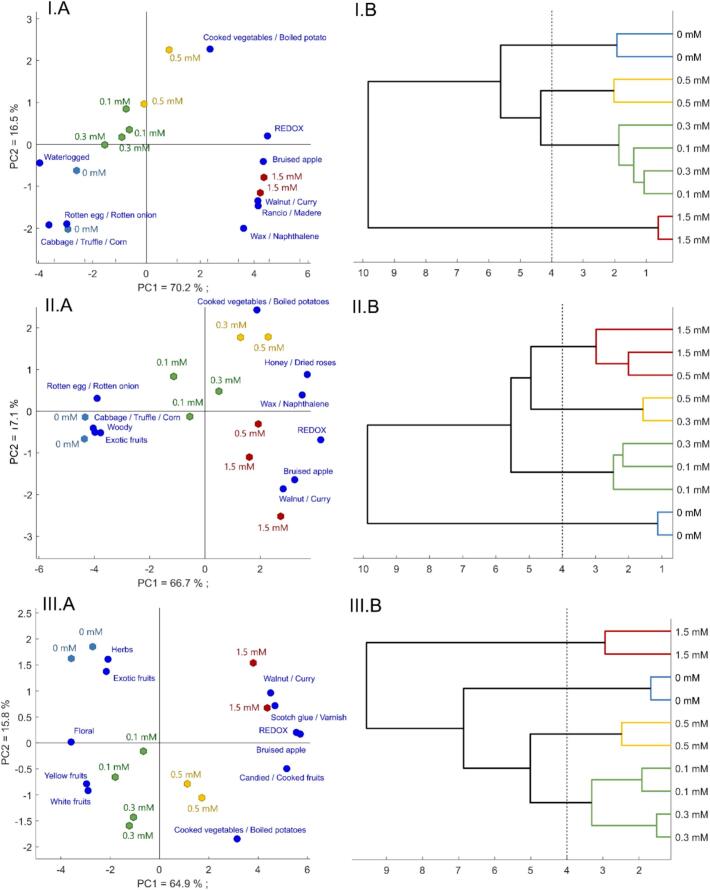
represents sensory analysis results obtained for 3 different wines from the 2019 vintage, I, II and III, respectively 2019-HCN, 2019-Ard and 2019-ChaJouan. A: PCA obtain with intensity of sensory descriptors which were significatively discriminant of modalities (Kruskal-Wallis, *p* < 0.05). Colors correspond to groups determined by HCA. B: HCA obtains for each studied wine.

Fig. 3 shows the results from the PCA and the cluster analysis for each wine. In detail, Fig. 3 shows the results obtained for 2019-HCN. PCA (Fig. 3I.AA) and HCA (3I.B) confirmed good repeatability and clear sample clustering. The control wine (0 mM H_2_O_2_) was characterized by cabbage/truffle/corn, waterlogged and rotten egg/rotten onion clearly suggesting reductive off flavors for the control wine. Low H_2_O_2_ concentrations (0.1 and 0.3 mM) led to the suppression of the reductive perception of wines without creating oxidative notes. These 2 modalities were not sensorially different Figure -IB). With higher H_2_O_2_ concentrations (0.5 mM) appeared first oxidative descriptors like cooked vegetables/boiled potatoes. The sample added with 1.5 mM H_2_O_2_ showed strong oxidized character described as bruised apple, walnut/curry, rancio/Madere and candied/cooked fruit (Table S2). HCA confirmed that 1.5 mM H_2_O_2_ addition was the most different modality compared to lower H_2_O_2_ addition.

The sensory results for the samples obtained from 2019-Ard wine are presented in Fig. 3II. The control wines, which showed strong reductive notes, appeared quite far apart from the other samples in the PCA (Fig. 3II.A and 3II.B). The sample added with 0.1 mM H_2_O_2_ did not show reductive notes while the addition of 0.3 and 0.5 mM H_2_O_2_ had similar sensory impact to that on 2019-HCN. These samples were characterized by honey/wilted roses, wax/naphthalene and cooked vegetables/boiled potatoes. 1.5 mM H_2_O_2_ samples were characterized by bruised apple and walnut/curry. Surprisingly, the Redox score determined by sensory panelists was significantly different between 0.3-,0.5-mM H_2_O_2_ modalities, and not between 0.5- and 1.5-mM modalities (Anova, Fisher test (LSD) *p* < 0.05, Table S3). Moreover, HCA show a lower accuracy for the discrimination of modalities, with higher variability. One duplicate of 0.3 mM H_2_O_2_ is closer to 0.1 mM H_2_O_2_ modalities, while the other one is closer to 0.5 mM H_2_O_2_. The second 0.5 mM H_2_O_2_ being closer to 1.5 mM H_2_O_2_. This result shows a low discrimination between the treated samples using the sensory descriptors. This result can be explained by the use of only duplicate, leading to less robust statistic treatments (Fig. 3IIB).

Results obtained for 2019-ChaJouan are shown in Fig. 3III. It appeared that the control wine was clearly discriminated from the treated samples and was characterized by fruity and floral descriptors (exotic fruits, herbaceous, white fruits, yellow fruits and floral). After the addition of 0.1 and 0.3 mM H_2_O_2_, no significant difference in terms of redox score was measured, and the wines were still characterized by fruity and floral descriptors. The 0.5 mM H_2_O_2_ replicates showed higher redox scores and were characterized by cooked vegetables/boiled potatoes descriptors. As for the other two wine matrices, the two 1.5 mM H_2_O_2_ samples were the most oxidized and were described by walnut/curry, bruised apple odors (Table S4). On the three studied wines, this matrix shows a lag phase during oxidation with a keeping of floral, white and exotic fruits descriptors until 0.3 mM H_2_O_2_ showing a better resistant against oxidation. Global parameters as antioxidant capacity (DPPH) and color change seem insufficient to study this phenomenon and further work are needs to understand deeper the compounds involved behind this resistance.

Across all matrices, low H₂O₂ concentrations (0.1 mM) effectively suppressed reductive off-odors without inducing oxidation. This is notable, as two of the three wines developed unexpected reductive characters during cold, anoxic storage, a condition known to promote sulfur-related faults. The transition from reduction to oxidation was progressive and wine specific. 2019-HCN and 2019-ChaJouan showed similar clustering at low concentrations (0.1, 0.3 mM) but diverged at higher doses. 2019-Ard showed more sensitivity to H₂O₂, with oxidative descriptors appearing as early as 0.3 mM, consistent with its lower resistance to oxidation. Cluster analyses confirmed that, first, lower oxidation levels were linked to descriptors like cooked vegetables / boiled potatoes and second, stronger oxidation (1.5 mM) yielded richer, deeper descriptors such as walnut/curry and bruised apple.

## 4 Conclusions

The objective of this study was to develop a quick and easy accelerated aging protocol for dry white wines that best reproduces the sensory and chemical effects of natural cellar aging. To achieve this, we tested and compared two accelerated aging methods: ultrasound irradiation and hydrogen peroxide (H₂O₂) addition. The ultrasound-based protocol failed to reproduce realistic oxidative evolution. Treated wines exhibited atypical off-odors such as “burnt plastic” and showed no significant changes in antioxidant capacity or sensory oxidation scores. These findings suggested that, under the conditions tested, ultrasound irradiation is not suitable for simulating oxidative aging in white wines. In contrast, the protocol based on controlled H₂O₂ addition showed promising results. It successfully produced wines with a range of increasing oxidative states, consistent with both chemical evolution (decreased antioxidant capacity, increased browning) and sensory changes (appearance of oxidative aroma descriptors such as bruised apple, walnut/curry, and cooked vegetables). REDOX scores increased with H₂O₂ dose, and antioxidant and colorimetric data showed strong correlation with sensory evolution. Interestingly, distinct wines exhibited distinct matrix-dependent evolutions during the accelerated aging process. While initial antioxidant capacity (Ec20) provided some insight into oxidative sensitivity, it was insufficient to predict long-term oxidative resistance. Our results align with recent findings suggesting that a more comprehensive evaluation including molecular profiling of nucleophilic antioxidants is necessary for accurately assessing oxidative stability. Therefore, the proposed H₂O₂ protocol should be considered a practical and reproducible oxidative-stress assay to induce and grade oxidation-type sensory profiles under standardized conditions, rather than a tool to study natural oxidative mechanisms or to predict bottle aging shelf life. The method can support comparative assessment of wine oxidative response across samples or conditions, but it does not establish mechanistic equivalence to natural bottle aging. Future work should include targeted/non-targeted chemical characterization and benchmarking against naturally bottle-aged counterparts, together with quantification of catalytic metals and oxygen kinetics, to evaluate pathway similarity and enable any predictive applications.

## CRediT authorship contribution statement

**Remy Romanet:** Writing – review & editing, Writing – original draft, Formal analysis, Data curation, Conceptualization. **Jordi Ballester:** Writing – review & editing, Validation, Supervision, Formal analysis, Data curation, Conceptualization. **Jérôme Mallard:** Validation, Methodology, Formal analysis. **Régis Gougeon:** Writing – review & editing, Supervision, Resources, Funding acquisition, Data curation, Conceptualization. **Maria Nikolantonaki:** Writing – review & editing, Writing – original draft, Validation, Supervision, Project administration, Methodology, Funding acquisition, Formal analysis, Data curation, Conceptualization.

## Declaration of competing interest

The authors declare that they have no known competing financial interests or personal relationships that could have appeared to influence the work reported in this paper.

## Data Availability

Data will be made available on request.

## Appendix A Supplementary data

Supplementary material Image 1
