# Supplementary material for: Accelerated oxidative aging tests for white wines: First correlation between physico-chemical and sensory oxidation responses
Source: Food Chem X. 2026 Feb 18;34:103687. doi: 10.1016/j.fochx.2026.103687 (PMC12936469; doi:10.1016/j.fochx.2026.103687)
Supplement: Supplementary file 1 — Supplementary material [file mmc1.docx]

**Accelerated oxidative aging tests for white wines: first correlation between physico-chemical and sensory oxidation responses**

Remy Romanet ^1,2^, Jordi Ballester ^3^, Jérôme Mallard ^3^, Régis Gougeon^1^, Maria Nikolantonaki ^1^*

^1^ Université Bourgogne Europe, Institut Agro, INRAE, UMR PAM, Institut Universitaire de la Vigne et du Vin-Jules Guyot, 21000 Dijon, France

^2^ DIVVA Platform, Institut Universitaire de la Vigne et du Vin-Jules Guyot, 21000 Dijon, France

^3^ Université Bourgogne Europe, Institut Agro, CNRS, INRAE, UMR CSGA, 21000 Dijon, France

Table S1 : Concentration in free sulfites in the studied wines determined by Sulfilyser measurement before accelerated aging.

| Sample | Free SO_2_ (mg/L) |
| --- | --- |
| 2018-HCB | ND^*^ |
| 2018-Bourg | 12.0 ± 0.0 |
| 2019-Ard | 5.7 ± 0.3 |
| 2019-HCN | 64.0 ± 0.9 |
| 2019-ChaJouan | 29.5 ± 0.7 |

*^*^ ND : No Data*


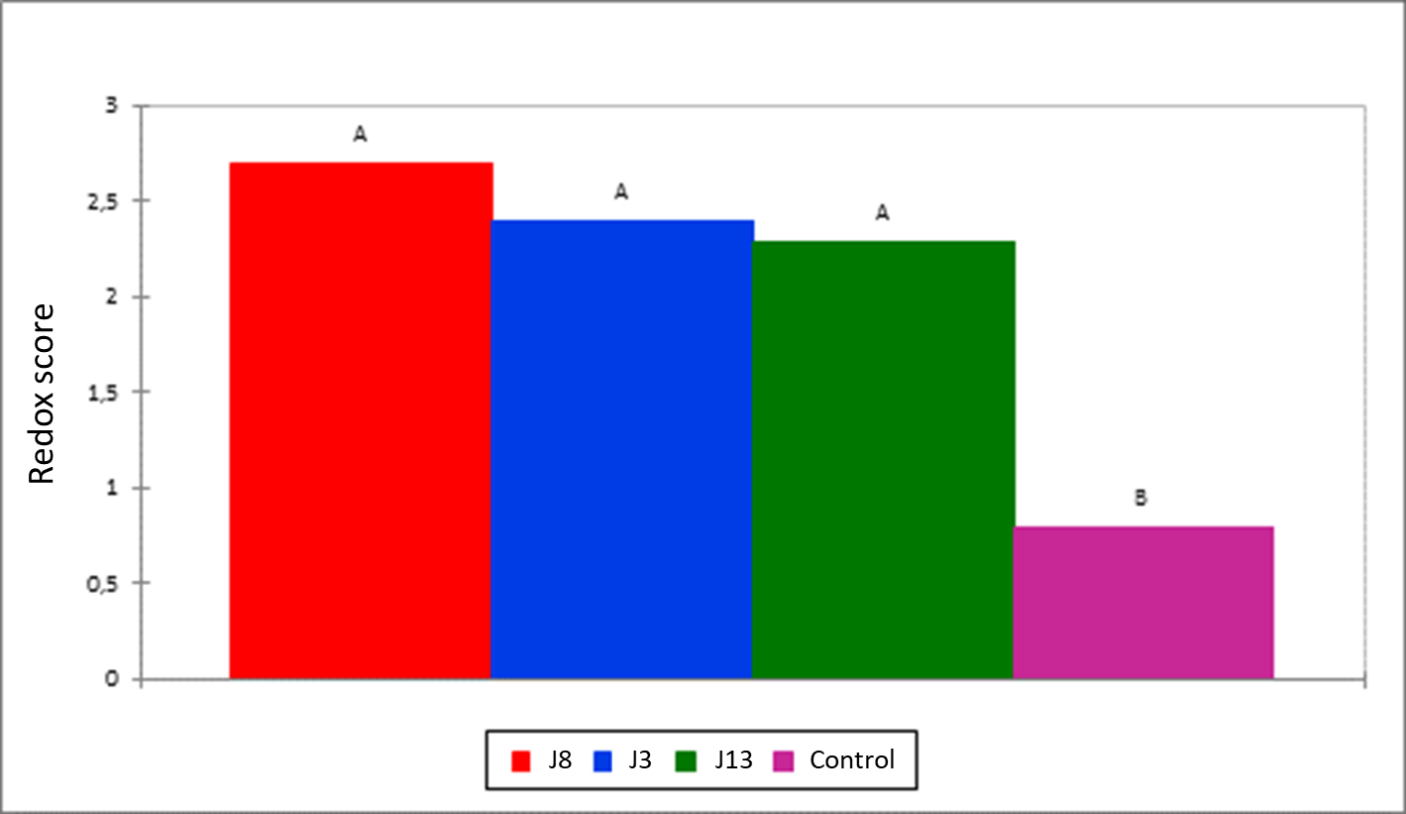


Figure S1 : Mean of Redox Score determinate by sensory analysis for 2018-Bourg after addition of 1 mM H_2_O_2_ and incubation of 3, 8 and 13 days. The control is untreated wine with H_2_O_2_.


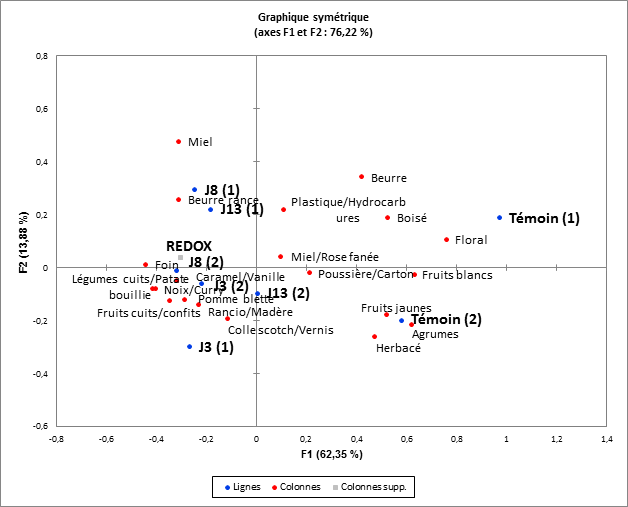


Figure S2 : Biplot obtain after sensory analysis for 2018-Bourg after addition of 1 mM H_2_O_2_ and incubation of 3, 8 and 13 days. Only sensory descriptors which are discriminant between samples have been used (Anova, p<0.05).


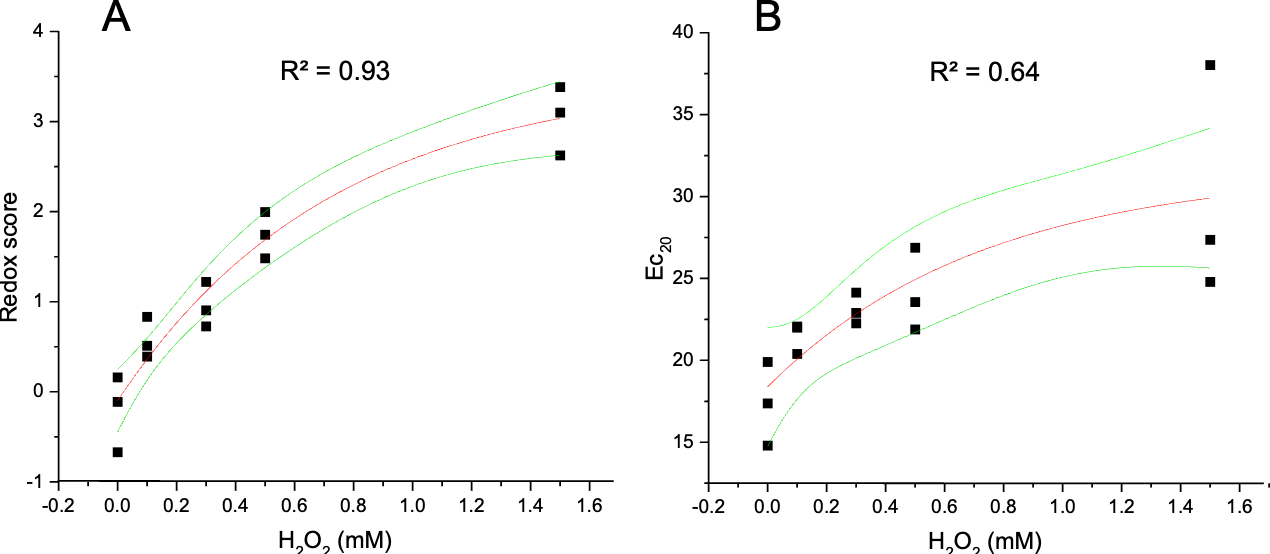


Figure S3: Evolution of the Redox score (A) and the antioxidant capacity (B) as a function of H_2_O_2_ concentration added, considering the pooled data from the 3 white wines from the 2019 vintage. The red line represents the regression line according to y=y_0_ + A*exp(R_0_.x) equation. Green dot lines represent the confidence level (95%) of the regression line.

Table S2 : Result of sensory analysis for 2019-HCN. Letters indicate significative differences between modalities for each descriptor (Kruskal-Wallis, Fisher test (LSD) p<0.05). For REDOX score: Anova, Fisher test (LSD) p<0.05.

Table S3: Result of sensory analysis for 2019-Ard. Letters indicate significative differences between modalities for each descriptor (Kruskal-Wallis, Fisher test (LSD) p<0.05). For REDOX score: Anova, Fisher test (LSD) p<0.05.

Table S4: Result of sensory analysis for 2019-ChaJouan. Letters indicate significative differences between modalities for each descriptor (Kruskal-Wallis, Fisher test (LSD) p<0.05). For REDOX score: Anova, Fisher test (LSD) p<0.05.
